# Characterization of a novel anti-PVRIG antibody with Fc-competent function that exerts strong antitumor effects via NK activation in preclinical models

**DOI:** 10.1007/s00262-024-03671-z

**Published:** 2024-03-30

**Authors:** Hongyu Xue, Zhimin Zhang, Li Li, Chenjuan Zhu, Keke Fei, Huijun Sha, Zhihai Wu, Xiaomin Lin, Feifei Wang, Shuaixiang Zhou, Xiya Deng, Yiming Li, Bingliang Chen, Yao Xiong, Kai Chen

**Affiliations:** 1 Department of Oncology, The First Affiliated Hospital of Soochow University, Suzhou, 215006 China; 2 Innovent Biologics (Suzhou) Co., Ltd., 168 Dongping Street, Suzhou Industrial Park, Suzhou, 215123 Jiangsu China

**Keywords:** Cancer immunotherapy, PVRIG, NK cells, Therapeutic antibody

## Abstract

Poliovirus receptor-related immunoglobulin domain-containing protein, or PVRIG, is a newly discovered immune checkpoint that has emerged as a promising target for cancer immunotherapy. It is primarily expressed on activated T and natural killer (NK) cells, and once engaged with its ligand, PVRL2, it induces inhibitory signaling in T cells, thereby promoting the functional exhaustion of tumor-infiltrating lymphocytes (TILs). Here, we characterized IBI352g4a, a novel humanized anti-PVRIG antibody with Fc-competent function, explored the mechanism of its antitumor activity in preclinical models, and systemically evaluated the contribution of FcrR engagement to PVRIG blockade-induced antitumor activity. IBI352g4a binds to the extracellular domain of human PVRIG with high affinity (*Kd* = 0.53 nM) and specificity, and fully blocks the interaction between PVRIG and its ligand PVRL2. Unlike other immune checkpoints, IBI352g4a significantly induced NK cell activation and degranulation, but had a minimal effect on T-cell activation in in vitro functional assays. IBI352g4a induced strong antitumor effect in several preclinic models, through in vivo mechanism analysis we found that both NK and T cells contribute to the antitumor effect, but NK cells play predominant roles. Specifically, a single dose of IBI352g4a induced significant NK cell activation in TILs, but T-cell activation was observed only after the second dose. Moreover, the Fc effector function is critical for both NK cell activation and treatment efficacy in vitro and in vivo. Our study, for the first time, demonstrates that both NK activation and FcrR engagement are required for antitumor efficacy induced by PVRIG blockade.

**Supplementary Information:**

The online version contains supplementary material available at 10.1007/s00262-024-03671-z.

## Introduction

PVRIG (also known as CD112R) is a member of the Ig superfamily of receptors that are expressed mainly on activated T cells and natural killer (NK) cells and bind to the single ligand PVRL2 (also known as CD112 or Nectin-2) [1]. PVRIG belongs to the Nectin and Nectin-like (Necls) family, which includes nine adhesion molecules involved in cell‒cell adhesion and other vital cellular processes. Cumulative evidence has revealed that the paired receptors that interact with ligands of this family have opposite functions in the control of T and NK cells. In parallel with TIGIT, PVRIG inhibits T and NK cell functions by either directly binding with its ligand or competing with DNAM-1 (CD226) for PVR and PVRL2 binding [2–5].

PVRL2, the only ligand of PVRIG, is highly expressed in multiple cancer types and has been associated with poor survival [6]. In addition, PVRL2 weakly binds to another inhibitory receptor, TIGIT, and is a ligand of the coactivating receptor DNAM-1. Among all these the receptors, PVRIG seems to be the predominant receptor in these networks, as it is reported to have the highest affinity for PVRL2 [7]. Unlike TIGIT, PVRIG is expressed on only activated NK cells and T cells, especially on cytotoxic lymphocytes, while it is expressed at low levels on Treg cells. However, inhibiting PVRIG using anti-PVRIG blockade significantly enhanced T-cell and NK cell cytotoxicity against several tumor cells in vitro [1]. Moreover, PVRIG blockade has been shown to reduce the tumor burden in a mouse model [8]. Taken together, these data suggest that targeting PVRIG may augment the immune response and achieve optimal antitumor efficacy.

Currently, several anti-PVRIG antibodies, either as bispecific Abs with TIGIT or monoclonal Abs with different Fc formats, have entered clinical trials. Antibody COM701 is the most advanced compound and, in combination with both anti-TIGIT and anti-PD-1 agents, is currently in phase 2 clinical trials. COM701 utilizes IgG4, an isotype with weak effector functions compared with IgG1 [9]. Moreover, two other benchmarks, SRF813 from surface oncology or NM1F from TG ImmunoPharma used IgG1 with full Fc function aiming to achieve better efficacy. [10]. These discrepancies, similar to those investigational anti-TIGIT Abs tested in the clinic [11], raise the following critical question: is an effective function-competent Fc format necessary for the antitumor effects of PVRIG blockade?

Here, we developed a humanized anti-PVRIG antibody with full Fc function, IBI352g4a. We characterized the affinity, blockade activity and functional activities both in vitro and in vivo. In addition, we systemically characterized the mechanism by which PVRIG blockade induces tumor killing via NK cell activation rather than T-cell activation and show that the effector function of competent Fc is necessary for the antitumor efficacy of PVRIG blockade in preclinical models.

## Materials and methods

### Cell lines and culture

K562 cells were purchased from American Type Culture Collection (ATCC) and cultured in Roswell Park Memorial Institute (RPMI) 1640 medium (Gibco) supplemented with 10% fetal bovine serum (FBS; Gibco) and 1% penicillin‒streptomycin (Gibco). GS-CHO stable cell lines overexpressing human PVRIG or cynomolgus monkey PVRIG (cynoPVRIG) were generated according to the manufacturer’s instructions using the GS Xceed Expression System (Lonza) and cultured in CD CHO medium (Gibco, Grand Island, NY, USA) supplemented with 75 μM MSX (Sigma).

### Antibody generation

IBI352g4a, a humanized IgG1 PVRIG antibody derived from the murine clone ch44G1 by hybridoma fusion, targets the human PVRIG extracellular domain. The variable regions of the heavy and light chains of ch44G1 were sequenced, and the murine framework regions were replaced by closely homologous human germline IgG sequences. The final form of the humanized anti-PVRIG monoclonal antibody, referred to as IBI352g4a, was selected as the final candidate. COM701, as BMK, is a humanized IgG4 PVRIG antibody that utilizes heavy and light chain sequences from CPA.7.021 in patent US 10,227,408 B2. All of the functional antibodies used in this study were purified in house (Innovent Biologics Co., Ltd., Suzhou, China) from HEK293 cells with either transient or stable expression unless indicated otherwise.

### Mice

B-NDG hIL15 mice were purchased from Beijing Biocytogen. BALB/c mice were purchased from Beijing Vital River Laboratory Animal Technology Co. Ltd. All animals were maintained under specific pathogen-free conditions in the Experimental Animal Center of Innovent Biologics Co., Ltd. (Suzhou, China). All animal-related experiments were approved by the Animal Use and Care Committee of Innovent Biologics.

### Binding affinity analysis

Binding affinities were determined with biolayer interferometry (BLI) using Fortebio OctetRED96e. The experimental antibody was loaded with AHC biosensors at the indicated concentrations. After washing, the sensors were dipped in buffer containing antigen at the indicated concentrations and then dissociated in SD buffer (sample dilution buffer: 50 ml of PBS + 0.1% BSA + 0.05% Tween-20). Data analysis was performed with FortéBiosoftware (Data Analysis 10.0).

### Cell-based binding assay

Human PVRIG and cynomolgus monkey PVRIG (cynoPVRIG) cell binding were measured via flow cytometry using stable PVRIG-overexpressing GS-CHO cells. For the PVRIG cell binding assay, human PVRIG-overexpressing GS-CHO cells and cynoPVRIG-overexpressing GS-CHO cells were incubated with serially diluted BMK, IBI352g4a or IgG, followed by staining with a PE-conjugated goat anti-human IgG Fc antibody (BioLegend, cat. # 366,904).

### Human PVRIG/PVRL2 blocking assay

Clear Flat-Bottom Immuno Nonsterile 96-well plates (Thermo Fisher Scientific, USA) were coated with 0.1 μg/mL recombinant human PVRIG protein (Acro Biosystems, Inc., Newark, Delaware, USA) in carbonate–bicarbonate buffer (Thermo Fisher Scientific, USA). After overnight incubation at 4 °C, the plates were blocked with PBS containing 1% BSA and 0.05% Tween-20 for 1 h. Serially diluted antibody and 2 μg/mL biotinylated human PVRL2 protein (Acro Biosystems, Inc., Newark, Delaware, USA) were added to the plates, which were subsequently incubated for 3 h at room temperature. After the samples were washed with PBS containing 0.05% Tween-20, biotinylated human PVRL2 protein was detected with streptavidin HRP (BioLegend, cat. # 405,210). The color reaction was initiated by adding TMB (Solarbio, Beijing #PR1200) and stopped with ELISA stop buffer (Solarbio, Beijing). The absorbance at 450 nm and 620 nm was measured with a microplate reader (Thermo, USA, Multiskan Fc).

### CynoPVRIG/PVRL2 blocking assay

Clear Flat-Bottom Immuno Nonsterile 96-well plates (Thermo USA 442404) were coated with 0.1 μg/mL recombinant cynoPVRIG protein (Acro Biosystems, Inc., Newark, Delaware, USA) in carbonate–bicarbonate buffer (Thermo Fisher Scientific, USA). After overnight incubation at 4 °C, the plates were blocked with PBS containing 1% BSA and 0.05% Tween-20 for 1 h. Serially diluted antibody and 2 μg/mL cynomolgus PVRL2 His protein (#90,206-C08H SinoBiological) were added to the plates and incubated for 3 h at room temperature. After washing with PBS containing 0.05% Tween-20, the protein expression of human PVRL2 was detected using an anti-6X His tag antibody (HRP) (Abcam, AB1187). The color reactions were initiated by adding TMB (Solarbio, Beijing #PR1200) and stopped with ELISA stop buffer (Solarbio, Beijing). The absorbance at 450 and 620 nm was measured with a microplate reader (Thermo, USA, Multiskan Fc).

### PVRIG expression in resting and activated peripheral blood mononuclear cells (PBMCs)

PVRIG expression in inactivated and activated PBMCs was determined. For resting PBMCs, PBMCs (AllCells Biotech Co., Ltd., Shanghai, China) were thawed, washed and resuspended in media (RPMI-1640 + 10% fetal calf serum). PBMCs were cultured without stimulation for 20 h, after which PVRIG expression was determined in different cell subpopulations of PBMCs via flow cytometry. For activated PBMCs, PBMCs were stimulated with Dynabeads™ human T-activator CD3/CD28 (Gibco, cat. # 11131D) and recombinant human IL-15 protein (R&D Systems, cat. # 247-ILB) for 24 h, 3 days, 6 days and 10 days. After culture, the cells were collected and stained for cell surface markers before flow cytometry.

### NK cell degranulation assay

PBMCs (AllCells) were thawed and treated with deoxyribonuclease I (Sigma) for 15 min at 37 °C. NK cells were isolated from PBMCs by using an EasySep™ Human NK Cell Enrichment Kit (Stemcell, Vancouver, BC, Canada) and cultured for 16 h. After overnight incubation at 37 °C, the NK cells were washed and cocultured with K562 cells at a 5:1 ratio for 4 h in the presence of 10 μg/mL BMK, 10 μg/mL IBI352g4a or 10 μg/mL control hIgG antibody. PE-conjugated anti-human 107a antibody (BioLegend, cat. # 328,608) and monensin (BioLegend, cat. # 420,701) were added to each well. After culture, the cells were stained with BV421 anti-human CD3 (BioLegend, cat. # 300,434) and AF488 anti-human CD335 (NKp46) (BioLegend, cat. # 331,938) and analyzed by flow cytometry.

### NK cell activation assay

NK cells isolated from PBMCs (AllCells) by an EasySepTM Human NK Cell Enrichment Kit (Stemcell) were cocultured with K562 cells at a 2:1 ratio for 16 h in the presence of 10 μg/mL BMK, 10 μg/mL IBI352g4a or 10 μg/mL control IgG antibody. After culture, the cells were stained with BV421-conjugated anti-human CD3 (BioLegend, cat. # 300,434), PE-conjugated anti-human CD335 (NKp46) (BioLegend, cat. # 331,908) and APC-conjugated anti-human CD137 (BioLegend, cat. # 309,810) antibodies and analyzed via flow cytometry.

### NK cell-mediated tumor cell death assay

NK cells isolated from PBMCs (AllCells) by an EasySepTM Human NK Cell Enrichment Kit (Stemcell) were cocultured with CellTrace Violet (CTV) Cell Proliferation Dye (Invitrogen, #C34557)-labeled K562 cells at a 5:1 ratio for 5 h in the presence of 10 μg/mL BMK, 10 μg/mL IBI352g4a or 10 μg/mL control IgG antibody. After culture, the cells were stained with propidium iodide solution (PI; BioLegend, cat. # 421,301), and the percentages of PI + CTV + dead K562 cells were analyzed via flow cytometry.

### Dose titration of PVRIG antibody induced cell death via NK killing

NK cells isolated from PBMCs (AllCells) were cocultured with K562 cells for 4 h in the presence of different doses of BMK, IBI352g4a or hIgG control antibody at a 5:1 ratio and analyzed via a CytoTox 96® Non-Radioactive Cytotoxicity Assay (Sigma‒Aldrich).

### Cytomegalovirus (CMV) specific T-cell activation assay

PBMCs from HLA-A2.1 + healthy donors were cultured with CMV pp65 peptide (NLVPMVATV, 495–503; HLA-A2.1-restricted, > 98% purity; synthesized by GL Biochem, Shanghai, China) in PVRL2 precoated 96-well flat-bottom plates in the presence of serial dilutions of the anti-PVRIG antibody. After the cells were cultured for 6 days in a 37 °C incubator with 5% CO_2_, the IFN-γ concentration in the culture supernatants was determined via ELISA.

### T-cell activation assay

OKT3 mAb (anti-human CD3; Thermo Fisher Scientific, cat. # MA1-10,175) at a concentration of 1 μg/mL was used to pretreat 96-well plates. Human T cells were negatively selected and purified from PBMCs (AllCells) using an EasySep™ Human T-Cell Isolation Kit (Stemcell, cat. # 17,951). T cells were added to the wells at 1.5 × 10^5^ cells/well and cultured for 24 h and 6 days in the presence of 10 μg/mL anti-PVRIG or control antibody. The control treatment was not precoated with the OKT3 mAb. After 24 h and 6 days, the cells were collected and stained for cell surface markers before flow cytometry analysis. The following antibodies were used in the flow cytometry analyses: AF488-conjugated anti-human CD4 (BioLegend, cat # 300,519), PerCP/Cyanine5.5-conjugated anti-human CD8a (BioLegend, cat. # 300,924), PE-conjugated anti-human CD69 (BioLegend, cat. # 310,906), BV421-conjugated anti-human CD25 (BioLegend, cat. # 356,114), and PE/Cyanine7-conjugated anti-human CD3 (BioLegend, cat. # 300,316).

### T-cell proliferation assay

OKT3 mAb (Thermo Fisher Scientific) at a concentration of 1 μg/mL was used to precoat 96-well plates. PVRL2 (Acro Biosystems, Inc., Newark, Delaware, USA) was also added to the wells. Human T cells were negatively selected from among the PBMCs (AllCells) and purified with an EasySep™ Human T-Cell Isolation Kit (Stemcell). T cells were CFSE labeled, added to wells at 3 × 10^5^ per well and cultured for 6 days in the presence of 10 μg/mL anti-PVRIG or control antibody. T-cell proliferation was assessed by flow cytometry analysis after 6 days of CFSE dilution.

### DAN-G/Claudin18.2 xenografts in a PBMC humanized model on B-NDG hIL15 mice

For the human NK cell-reconstituted xenograft model, B-NDG huIL-15 mice were inoculated subcutaneously with 1 × 10^6^ DAN-G/Claudin18.2 cells on day 0. Mice were intravenously (i.v.) injected with 4 × 10^6^ human PBMCs on day 5. Mice were randomly grouped on day 7 and intraperitoneally injected with hIgG (10 mg/kg), BMK (10 mg/kg), or IBI352g4a (10 mg/kg) on days 7, 10, 13, 17, 20, and 24. Tumor volume and mouse body weights were measured twice weekly. Mice were euthanized once their tumor volume reached 2000 mm^3^.

### DAN-G/Claudin18.2 xenografts in PBMC humanized model on NOG mice

NOG mice were i.v. injected with 4 × 10^6^ human PBMCs on day 0 and subcutaneously inoculated with 1 × 10^6^ DAN-G/Claudin18.2 cells on day 3. Mice were randomly grouped into two groups when the mean tumor volume reached 80 mm^3^. Anti-PVRIG or hIgG was intraperitoneally administered at 10 mg/kg on days 5, 8, 11, 15, 18, and 22. Tumor and mouse body weights were measured twice weekly. Mice were euthanized once their tumor volume reached 2000 mm^3^.

### In vivo mechanism of action (MoA) study using a CT26 tumor model

To study the MoA of the anti-PVRIG antibody in a mouse tumor model, BALB/c mice were subcutaneously inoculated with CT26 tumor cells on day 0. Mice were randomly grouped when the mean tumor volume reached 80 mm^3^. Mice were treated with hIgG (10 mg/kg) or anti-PVRIG (10 mg/kg) on day 7 (first dose) or day 11 (second dose). Tumor-infiltrating lymphocytes were analyzed by flow cytometry on days 8 and 12.

### In vivo mechanistic study using NK cell or T-cell depletion

To deplete NK cells or CD8^+^ T cells, mice were given an intraperitoneal injection of 2.5 mg/kg Asioa GM1 polyclonal antibody (Invitrogen, cat. # 16-6507-39) or 10 mg/kg InvivoMAb to CD8β (Bio X cell, cat. # BE0223) 24 h before CT26 tumor cell inoculation, after which the cell depletion antibodies were injected once every week before anti-PVRIG or IgG treatment.

### PBMC sample analysis by FACS

The cells were collected and preincubated with a LIVE/DEAD™ Fixable Near-IR Dead Cell Stain Kit (Invitrogen) and Fc receptor blocking agent (BioLegend, cat. # 422,302) in PBS for 15 min at 4 °C before being stained with a fluorochrome-conjugated anti-human antibody. The following antibodies were used in the flow cytometry analyses: AF700 anti-human CD3 (BioLegend, cat. # 300,324), BV711 anti-human CD8a (BioLegend, cat. # 301,044), PE anti-human CD4 (BioLegend, cat. # 300,508), FITC anti-human CD14 (BioLegend, cat. # 325,604), PerCP/Cyanine5.5 anti-human CD56 (BioLegend, cat. # 318,322), BUV496 mouse anti-human CD16 (BD, cat. # 612,945), PE/Cyanine7 anti-human CD19 (BioLegend, cat. # 302,216) and APC anti-human CD112R (PVRIG) (BioLegend, cat. # 301,506).

### TILs sample analysis by FACS

The tumors from CT26 tumor-bearing mice were collected, dissociated into single-cell suspensions using a tumor dissociation kit (Miltenyi Biotec, cat. # 130-096-730) and filtered through a cell strainer (Corning). Red blood cells were lysed using 1 × RBC lysis buffer (Invitrogen, cat. # 00-4333-57). Prior to fluorochrome-conjugated anti-human antibody labeling, the cell suspensions were preincubated with reagents from a LIVE/DEAD™ Fixable Near-IR Dead Cell Stain Kit (Invitrogen) and Fc receptor blocking agent (BioLegend, cat. # 101,320) in PBS for 15 min at 4 °C and then stained with a mixture of surface fluorescent Abs in FACS buffer for 30 min at 4 °C. For intracellular staining, the cells were washed and fixed for 30 min at 4 °C with intracellular fixation and permeabilization buffer (eBioscience, cat. # 00–5523-00). The fixed cells were stained with antibodies against Granzyme B, IFN-γ and perforin. The following antibodies were used for the flow cytometry analysis: FITC-conjugated anti-mouse NK1.1 (Invitrogen, cat. # 11–5941-82), PerCP/Cyanine5.5-conjugated anti-mouse CD8a (Invitrogen, cat. # 45–0081-80), BUV496-conjugated anti-mouse CD4 (BD, cat. # 741,051), PE/Cyanine7-conjugated anti-mouse CD45 (BioLegend, cat. # 103,114), AF647-conjugated anti-mouse CD107a (BioLegend, cat. # 121,610), BV421-conjugated anti-human/mouse Granzyme B (BioLegend, cat. # 396,414), BV785-conjugated anti-mouse IFN-γ (BioLegend, cat. # 505,837) and PE-conjugated anti-human perforin (BioLegend, cat. # 154,406).

All of the flow cytometry data were acquired using a BD FACS Symphony A3 (BD Biosciences) and were analyzed using FlowJo software (FlowJo, LLC, Ashland, Oregon, USA).

### Statistical analysis

Statistical analyses were performed in GraphPad Prism (La Jolla, USA) using the appropriate tests (unpaired two-tailed t test, two-way ANOVA), as indicated in the figure legends. *p* < 0.05 was considered to indicate statistical significance.

## Results

### Characterization of the PVRIG-binding affinity and ligand blocking activity of IBI352g4a

**Fig. 1:**
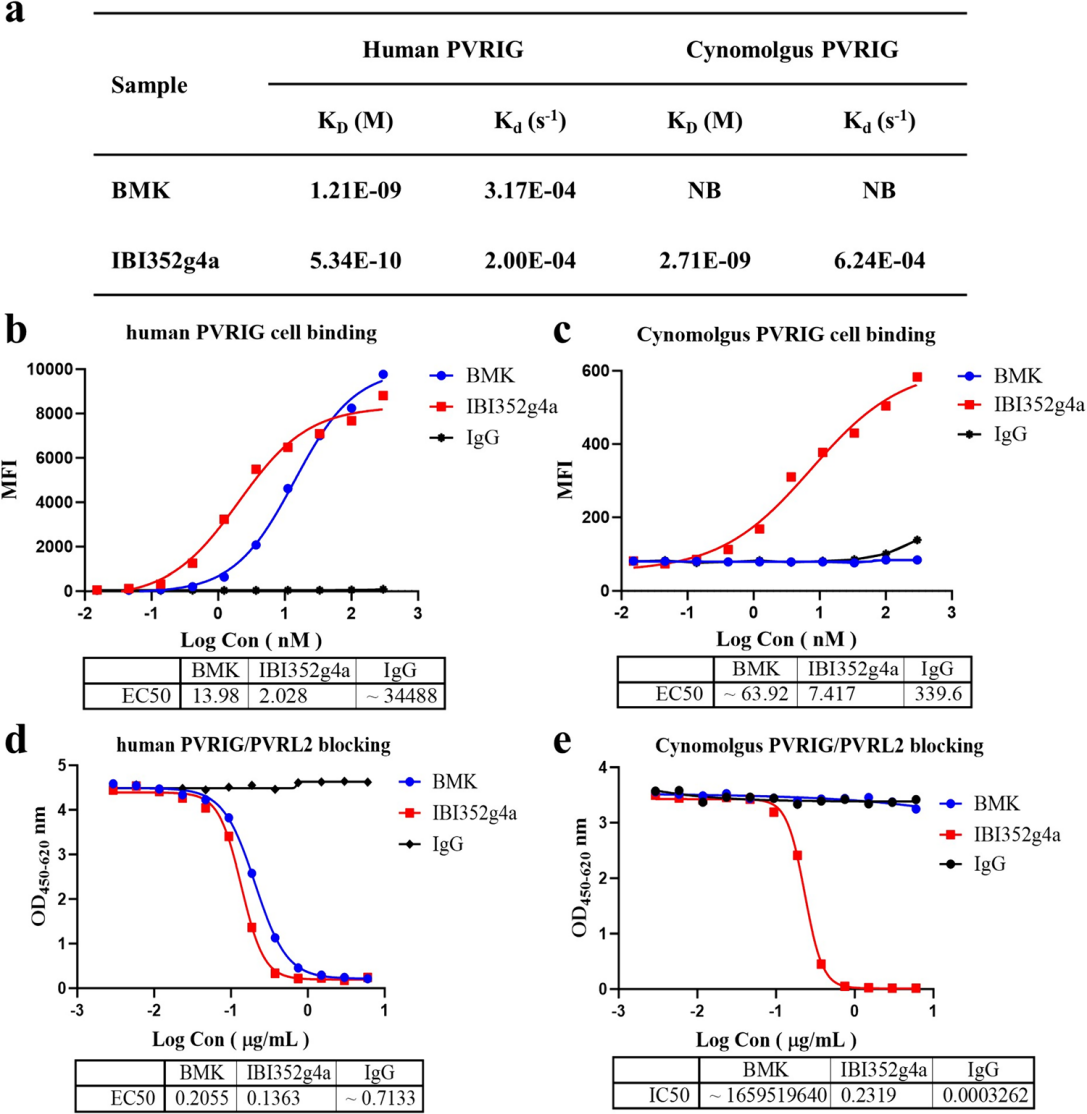
Binding affinity and ligand blocking activity of IBI352g4a. **a** Affinity of BMK and IBI352g4a for human PVRIG and cynoPVRIG assayed using FortéBio. **b** Human PVRIG cell-based binding was conducted for BMK and IBI352g4a using the GS-CHO/hPVRIG stable cell line. **c** CynoPVRIG cell-based binding was conducted for BMK and IBI352g4a using the GS-CHO/cynoPVRIG stable cell line. **d** ELISA-based human PVRIG/PVRL2 blocking assay. Biotinylated human PVRL2 cells were incubated with increasing amounts of IBI352g4a or BMK in precoated human PVRIG plates. After incubation and washing, biotinylated human PVRL2 was detected via streptavidin (HRP). **e** ELISA-based cynoPVRIG/PVRL2 blocking assay. CynoPVRL2 protein (his tag) was incubated with increasing amounts of IBI352g4a or BMK in precoated cynomolgus PVRIG plates. After incubation and washing, the expression of cynomolgus monkey PVRL2 was detected with an HRP-conjugated antibody

Fourteen clones against PVRIG were generated by hybridoma fusion. After several rounds of primary screening based on binding affinity and blocking activity (data not shown), IBI352g4a was selected as the molecule of interest and was humanized by complementarity-determining region (CDR) grafting. As shown in Fig. 1a, IBI352g4a strongly binds to human PVRIG with a Kd of 0.53 nM according to the BLI assay. This high binding affinity was further confirmed by a cell binding assay using a human PVRIG-overexpressing GS-CHO cell line (CHO-huPVRIG) with an EC50 of 2.03 nM (Fig. 1b). In addition, IBI352g4a completely blocks the interaction between PVRIG and its ligand PVRL2, with an IC50 of 0.94 nM according to an ELISA-based ligand competition assay, as shown in Fig. 1d.

Moreover, the binding ability of IBI352g4a with cynoPVRIG was also determined; the Kd of IBI352g4a was 2.71 nM for cynoPVRIG, and the EC50 of cell binding activity was 7.42 nM based on overexpressed cynoPVRIG CHO-S cell lines (Fig. 1a, c and e). These data demonstrated that IBI352g4a bound to both human and cynoPVRIG proteins with similar affinities.

### Functional characterization of IBI352g4a in vitro

**Fig. 2:**
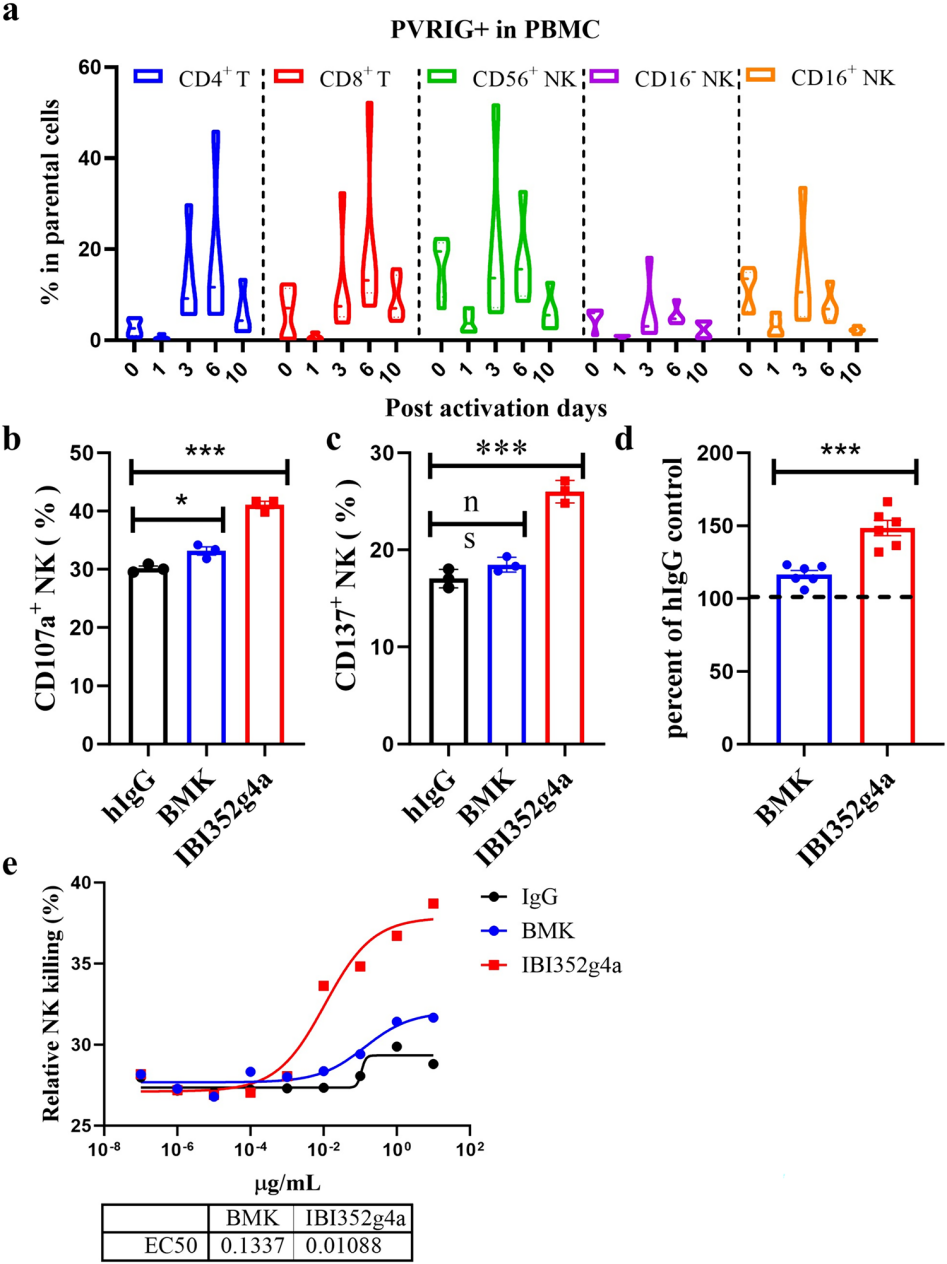
IBI352g4a induces NK function in vitro in a cell-based assay. **a** The expression of PVRIG in PBMCs on different days was analyzed after human T-activator CD3/CD28 Dynabeads and IL-15 stimulation by FACS. **b** NK cells were isolated from PBMCs and cultured for 16 h. After overnight incubation at 37 °C, the NK cells were washed and then cocultured with K562 cells at a 5:1 ratio for 4 h in the presence of BMK, IBI352g4a or the hIgG control antibody. PE-conjugated anti-CD107a antibody was added to each well. The expression of CD107a in NK cells was analyzed by FACS. **c** NK cells isolated from PBMCs were cocultured with K562 cells at a 2:1 ratio for 16 h in the presence of BMK, IBI352g4a or the hIgG control antibody. The expression of CD137 in NK cells was analyzed by FACS. **d** NK cells isolated from PBMCs were cocultured with CTV-labeled K562 cells at a 5:1 ratio for 5 h in the presence of BMK, IBI352g4a or the hIgG control antibody. CTV^+^ PI^+^ dead K562 cells were analyzed by FACS. **e** Cytotoxicity of NK cells against K562 cells in the presence of different doses of BMK, IBI352g4a or hIgG control antibody at a 5:1 ratio was analyzed. For **b–d**, the data were analyzed by an unpaired Student’s t test; ns, not significantly different; *, *P* < 0.05; ***, *P* < 0.001. The data are representative of three independent PBMC donors. The error bars represent the standard error of the mean (SEM)

As PVRIG is reported to be broadly expressed on activated T and NK cells [1, 7, 8, 12], we first examined its expression profile in PBMCs. Like in a previous study, in resting PBMCs, PVRIG was detectable at low levels in only CD8^+^ T cells and CD56^+^CD16^+^ NK cells, while CD19^+^ B cells, CD14^+^ monocytes, CD4^+^ T cells and CD56^+^CD16^−^ NK cells did not express surface PVRIG (Fig. S1). Then, once PBMCs were activated with CD3/CD28 beads and IL-15, the expression of PVRIG on both T and NK cells immediately decreased to undetectable levels, increased after three to six days, and subsequently decreased to baseline expression on day 10 (Fig. 2a). Unlike PD-1 and TIGIT, PVRIG was expressed at similar levels on both activated T cells and NK cells. Taken together, the different expression profiles of PVRIG, TIGIT and PD-1 suggest that these three immune checkpoint molecules may have different immunological functions.

Next, as PVRIG is expressed equally on both T cells and NK cells, several assays were used to assess the effects of IBI352g4a on naturally derived T cells, NK cells or whole PBMCs.

A coculture system of purified NK cells with the myelogenous leukemia cell line K562, which expresses high levels of PVRL2, was used for the study of PVRIG mAb-mediated NK function. NK cell activation was determined by measuring the NK cell degranulation marker CD107a via FACS after 4 h of coculture (Fig. 2b) and the activation marker CD137 after 16 h of coculture (Fig. 2c). The percentages of both CD107a^+^ and CD137^+^ total NK cells (gating from NKp46^+^ CD3^−^ cells) were significantly increased upon IBI352g4a treatment. Moreover, the NK-mediated killing effect of IBI352g4a was also measured via the same system. K562 cells were labeled with cell trace violet (CTV) and cocultured with freshly derived NK cells at a ratio of 5:1. After 5 h of treatment with antibodies, a significant increase in the number of PI and CTV double-positive cells was detected in the anti-PVRIG mAb-treated cells compared with the IgG-treated cells, suggesting that IBI352g4a induced greater NK cell-mediated killing than BMK (Fig. 2d). Moreover, the dose‒response relationship of IBI352g4a-induced tumor cell death was measured in the K562/NK coculture system. IBI352g4a and control antibodies at doses ranging from 10 ng/ml to 10 µg/ml were added to the coculture system. After 16 h of culture, IBI352g4a significantly induced greater NK-mediated tumor cell death than both IgG treatment and BMK, with an EC50 of 0.075 nM, as shown in Fig. 2e. These data suggest that IBI352g4a significantly induces tumor cell death via NK cell activation.

In parallel, how IBI352g4a affects T cells was also examined via several assays. Surprisingly, unlike what we observed in NK cells, little T-cell activation was observed upon anti-PVRIG mAb treatment. To conduct the human cytomegalovirus (CMV)-specific T-cell activation assay, PBMCs that recognize the CMV peptide were seeded in precoated PVRL2 plates in the presence of CMV and an anti-PVRIG antibody at various dosages. IFN-γ secretion was measured after 6 days of treatment as a readout of T-cell activation. Sintilimab (an anti-PD-1 antibody) served as a positive control and promoted IFN-γ production by CMV-specific T cells in a dose-dependent manner, while IBI352g4a mediated IFN-γ production in a manner similar to that of the isotype control (Fig. S2a). Next, we asked whether anti-PVRIG mAbs affect T-cell activation in addition to affecting CD3 and CD28 expression. Several different activation conditions for freshly derived T cells were examined in the presence of PVRIG mAbs, such as CD3/CD28 beads, coating CD3 soluble CD28 and coating CD3/CD28. Among these conditions, PVRIG mAbs failed to upregulate the expression of CD25 and CD69 on both CD4^+^ T and CD8^+^ T cells (Figure S2b). Moreover, PVRIG mAbs could not promote T-cell proliferation or CD4^+^ or CD8^+^ T-cell proliferation in the presence of anti-CD3 or anti-CD28 beads (Fig. S2c). Besides T cells activation and proliferation, we also detected the direct effect of PVRIG mAbs on cytotoxic T cells, consistent with other data, IBI352g4a failed to elicit any cytotoxic mediators, such as IFN-r, Granzyme B and Perforin at various dose from 0.01 to 10 ug/mL at different incubation time from 24 to 120 h (Fig. S2d), and induced direct T cells-mediated tumor killing effect (Fig. S2e). These data suggest that the anti-PVRIG mAb has little effect on T-cell function, activation or proliferation.

Taken together, the results of the in vitro functional assays showed that IBI352g4a strongly promoted NK cell activation and NK cell-mediated tumor cell killing but had minimal effects on T cells in vitro.

### Antitumor efficacy of IBI352g4a in humanized tumor mouse models

**Fig. 3:**
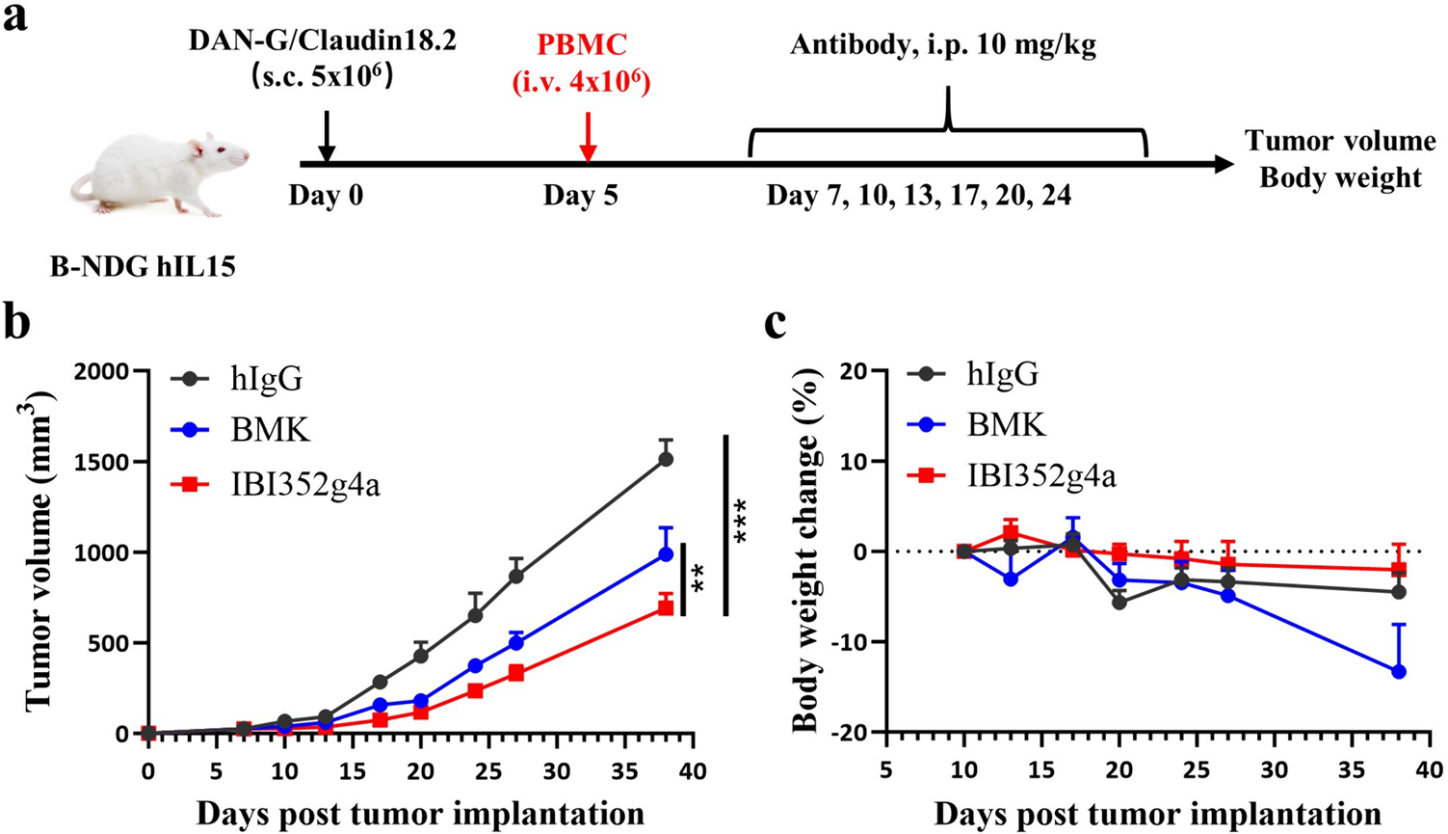
IBI352g4a has potent antitumor efficacy in humanized B-NDG hIL15 mice bearing DAN-G/Claudin18.2 tumors. **a** B-NDG hIL15 mice were subcutaneously inoculated with DAN-G/Claudin18.2 tumor cells on day 0. PBMCs were i.v. injected on day 5. Mice were grouped randomly and then intraperitoneally treated with hIgG (10 mg/kg), BMK (10 mg/kg), or IBI352g4a (10 mg/kg) twice a week starting on day 7. (**b**) Tumor size and (**c**) body weight change (%) in mice measured at various time points. The data are shown as the mean ± SEM. For **b**, comparisons between each treatment were performed using two-way ANOVA; **, *P* < 0.01; ***, *P* < 0.001

Next, we further explored the in vivo antitumor activity of IBI352g4a in various preclinical models. Considering that common humanized NOG mice exhibit insufficient immune system development and immune cell diversity and function, especially for NK cells, the applicability of these mice in human cancer treatment experiments may be limited. Recently, several next-generation humanized mouse models that express human IL-15 have been developed to promote human NK cell development [13]. Thus, in this study, two different humanized mouse models with different mouse backgrounds, NOG and B-NDG hIL15, were generated for in vivo efficacy evaluation. Consistent with the findings of other published data [13], only T cells (huCD3^+^/muCD45^+^) were detected in the peripheral blood of NOG-reconstituted mice, while in PBMC engrafted B-NDG hIL15 mice model, overall higher immune cell (both NK and T) frequencies are detected after 7 or 14 days engrafting, both T cells and NK cells exhibited greater proportion than that in NOG mice (Fig. S3a). IBI352g4a showed strong antitumor activity against DAN-G-18.2 human gastric tumors in B-NDG hIL15 mice (Fig. 3), which is a humanized model with both NK and T cells reconstitution, but less efficacy against tumors in NOG mice, which is a T-cell-only reconstitution model (Fig. S3b,c). These data suggested that both NK and T cells are required for the in vivo antitumor effect induced by IBI352g4a, only T cells are not sufficient enough for the antitumor effect for PVRIG blockade.

### PVRIG blockade activated tumor-infiltrated NK cells before T cells in tumor-bearing mice

**Fig. 4:**
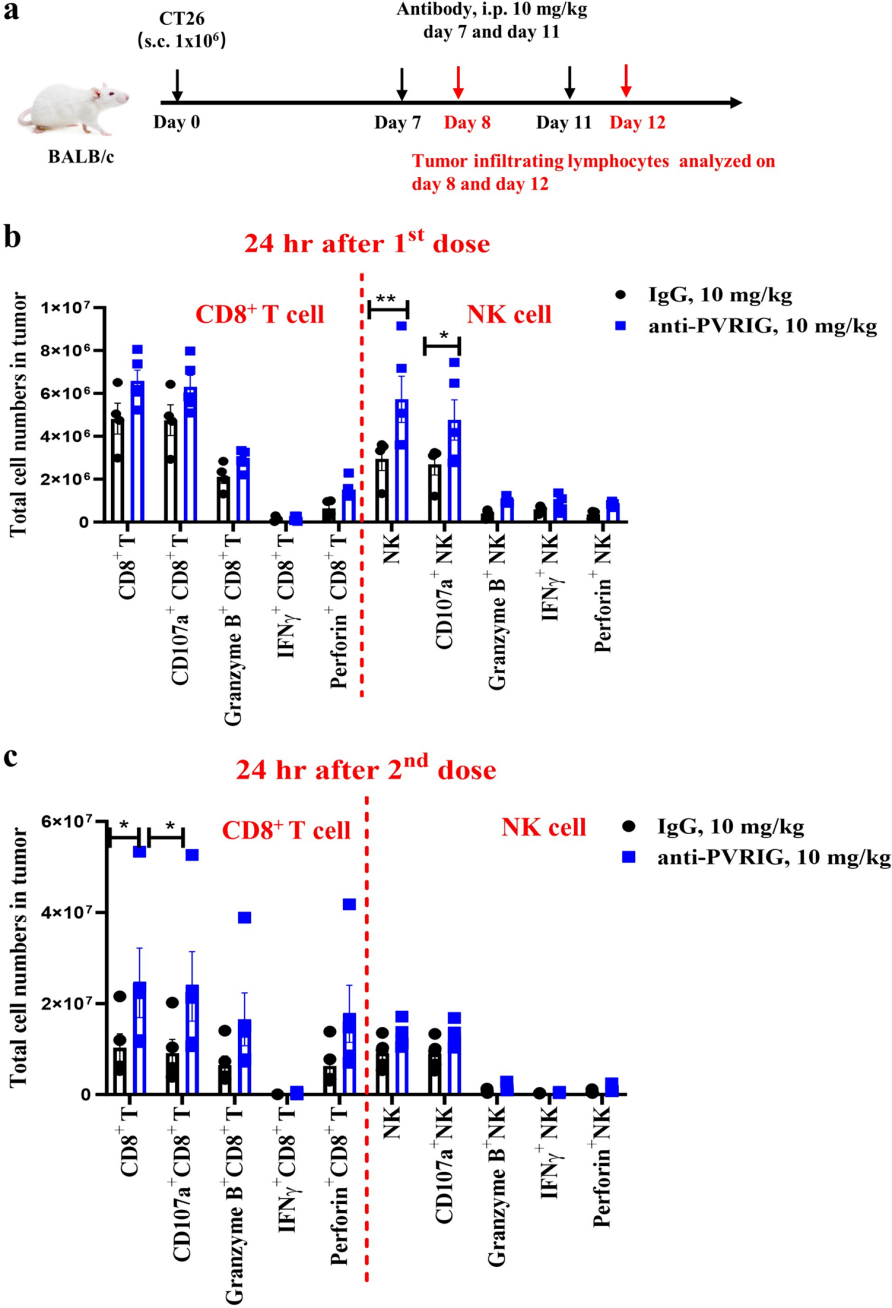
PVRIG blockade first activated tumor-infiltrated NK cells and subsequently activated T cells in tumor-bearing mice. **a** BALB/c mice were subcutaneously inoculated with CT26 tumor cells on day 0. Mice were randomly grouped and then treated with hIgG (10 mg/kg) or anti-PVRIG (10 mg/kg) on day 7 (first dose) or day 11 (second dose). TILs were analyzed by flow cytometry at 24 h after treatment **(b, c)**. Total CD8^+^ T cells, NK cells, CD107a^+^ CD8^+^ T cells and NK cells, Granzyme B^+^ CD8^+^ T cells and NK cells, IFNγ^+^ CD8^+^ T cells and NK cells, and perforin^+^ CD8^+^ T cells and NK cells in tumors were measured on day 8 **(b)** and day 12** (c)**. The data are shown as the mean ± SEM. Statistical significance was determined using a t test; *, *P* < 0.05; **, *P* < 0.01

To further explore the possible mechanisms underlying the therapeutic effects of the anti-PVRIG antibody in vivo, a surrogate PVRIG mAb was employed for the MoA study (Fig. 4a). The functional status of TILs in CT26 tumor-bearing mice treated with the anti-PVRIG mAb and those treated with the control antibody was determined by FACS after the first and second doses. Tumor-bearing mice treated with the anti-PVRIG mAb presented a significantly greater number of NK cells (NKp46^+^ in CD45^+^ cells) and upregulation of CD107a, while the number of total CD8^+^ T cells remained unchanged after the first dose (Fig. 4b). However, after the 2nd dose, the total number of CD8^+^ T cells in the tumor environment significantly increased, and the expression levels of Granzyme B, CD107a, and perforin increased. Moreover, the percentages of NK cells and levels of activation markers remained similar between the treated and untreated groups (Fig. 4c). Collectively, these results demonstrated that blocking PVRIG elicited potent NK cell activation first, followed by T-cell activation, resulting in potent systemic antitumor immunity against tumors and tumor growth delay via both cytotoxic TIL activation and exhaustion prevention.

**Fig. 5:**
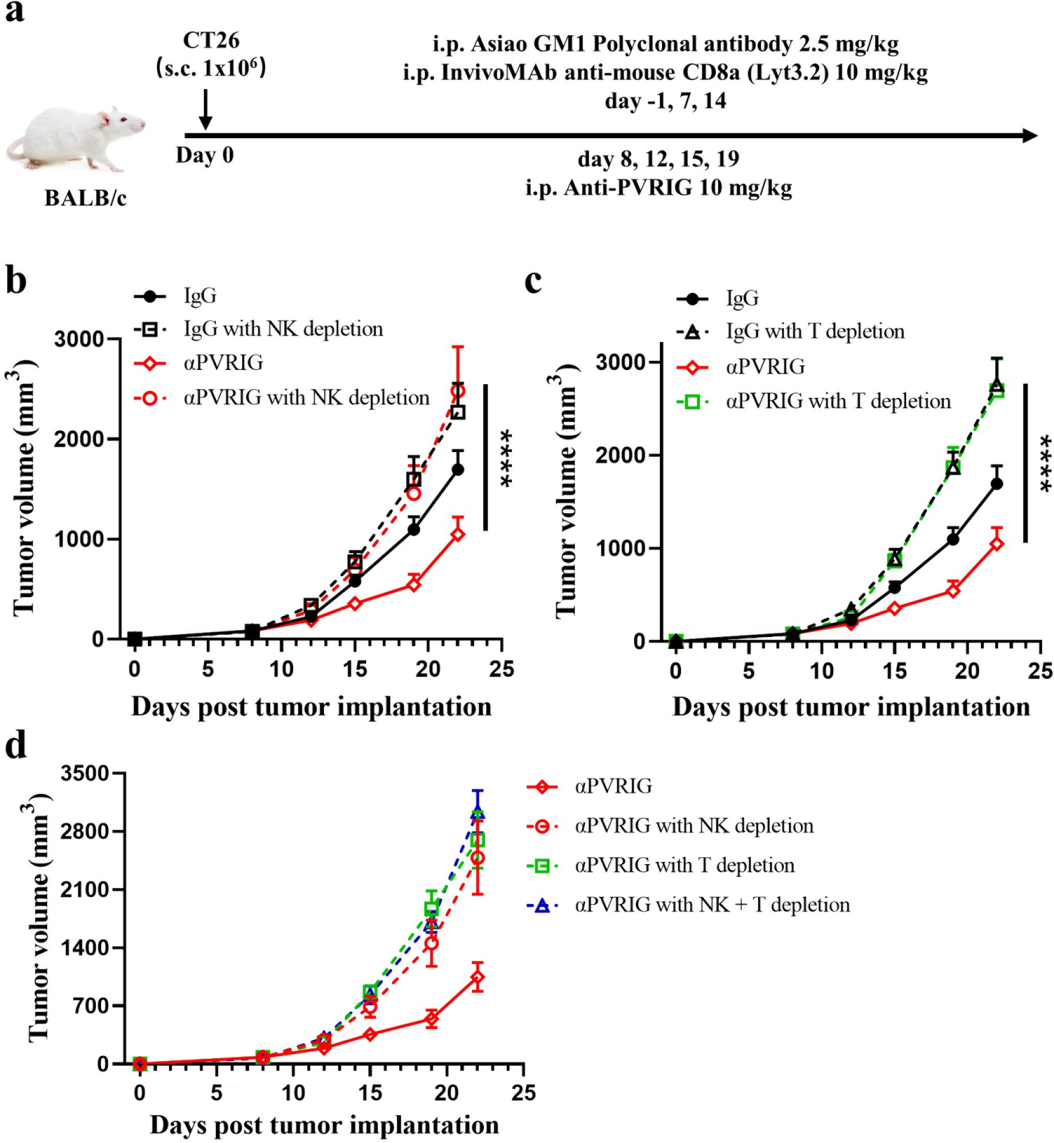
NK cells and CD8^+^ T cells both contributed to the antitumor efficacy of the anti-PVRIG antibody. **a** Experimental schedule for the CT26 tumor model used in **b**–**d**. Mice were intraperitoneally injected with IgG, anti-PVRIG, IgG combined with Asioa GM1 polyclonal antibody, IgG combined with anti-mouse CD8a antibody, anti-PVRIG combined with Asioa GM1 polyclonal antibody, anti-PVRIG combined with anti-mouse CD8a antibody or anti-PVRIG combined with Asioa GM1 polyclonal antibody and anti-mouse CD8a antibody at various time points after subcutaneous injection on day 0. **b**–**d** Tumor sizes were measured at various time points in mice subjected to different treatments. The data are shown as the mean ± SEM. Statistical significance was determined using two-way ANOVA; ****, *P* < 0.0001

To further investigate the roles of NK cells and CD8^+^ T cells in the therapeutic effect of PVRIG blockade, NK cells and/or CD8^+^ T cells were depleted in tumors by treating the mice with depleting antibodies against NK1.1 or CD8, respectively. Successful depletion of NK cells or CD8^+^ T cells was verified by FACS, as shown in Fig. S4. Although the anti-PVRIG mAb significantly inhibited tumor growth, NK cell depletion abolished this effect in tumor-bearing mice (Fig. 5a). Moreover, a similar trend was observed for T-cell depletion in the anti-PVRIG mAb treatment group (Fig. 5b). Consistent with the findings of the TIL profile in the mechanistic study, both NK and T cells, innate and adaptive immunity contributed to the therapeutic effect of the anti-PVRIG mAb (Fig. 5c). However, considering the limited efficacy of IBI352g4a in the PBMC engraftment model on NOG mice, (Fig. S3c), which is a T-cell-only reconstitution model, the importance of NK cells to the anti-PVRIG-induced antitumor effect was demonstrated.

### IBI352g4a induced antitumor effects in an Fc-dependent manner both in vitro and in vivo

**Fig. 6:**
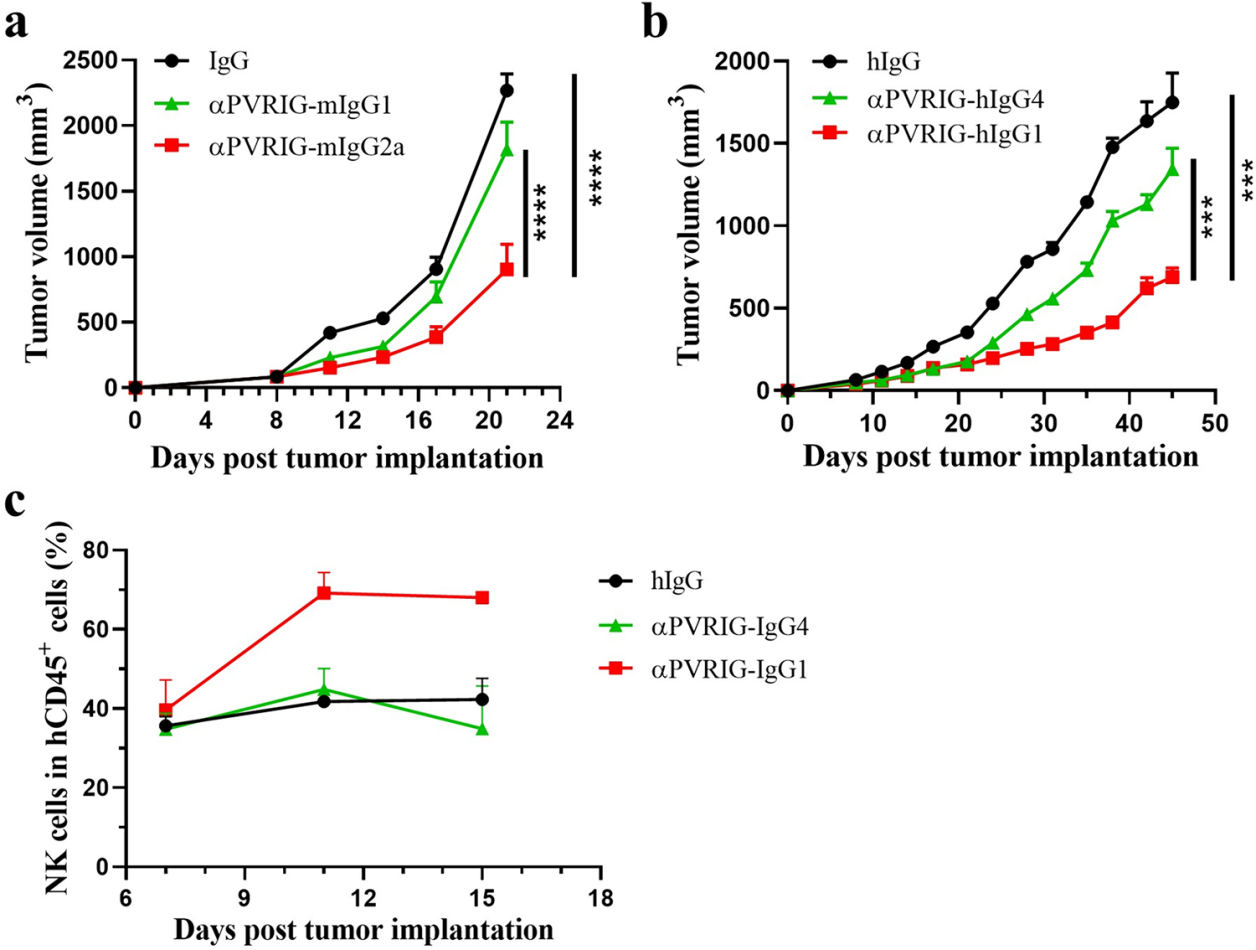
Anti-PVRIG mIgG2a shows stronger antitumor efficacy than does anti-PVRIG mIgG1. **a** BALB/c mice were subcutaneously inoculated with CT26 tumor cells on day 0. Mice were randomly grouped and then intraperitoneally treated with IgG (10 mg/kg), anti-PVRIG mAb (10 mg/kg) or anti-PVRIG mAb (10 mg/kg) twice a week starting on day 8. Tumor size and mouse weight were measured at various time points. (**b**, **c**) B-NDG hIL15 mice were subcutaneously inoculated with DAN-G/Claudin18.2 tumor cells on day 0. PBMCs were i.v. injected on day 5. Mice were randomly grouped and then intraperitoneally treated with IgG (10 mg/kg), anti-PVRIG hIgG4 (10 mg/kg) or anti-PVRIG hIgG1 (10 mg/kg) twice a week starting on day 7. (**b**) Tumor size and (**c**) percentage of NK cells (%) among the CD45^+^ cells in the mice measured at various time points. The data are shown as the mean ± SEM. Statistical significance was determined using two-way ANOVA; ***, *P* < 0.001; ****, *P* < 0.0001

Finally, we evaluated the relative contribution of Fc functions to the antitumor activity of PVRIG blockade antibodies. First, we performed mouse efficacy studies using CT26 tumor BALB/c model mice using a surrogate PVRIG mAb with mouse IgG1 or mouse IgG2a. As shown in Fig. 6a, biweekly dosing of the PVRIG murine antibody with muIgG2a Fc at 10 mg/kg significantly inhibited tumor growth; in comparison, the antibody with muIgG1 Fc was less effective. Next, the efficacy of anti-PVRIG mAbs with different human IgG Fc formats was detected in the DNG-18.2 humanized B-NDG hIL15 mouse model, as shown in Fig. 6b. Compared with huIgG4 Fc, the anti-PVRIG mAb with huIgG1 Fc achieved greater efficacy. Moreover, more NK cells (Fig. 6c) were detected in the peripheral blood of IgG1-treated mice than in that of IgG4-treated mice. Taken together, these data suggest that Fc effector functions play a critical role in the antitumor efficacy of the PVRIG antibody in vivo.

## Discussion

In this report, we showed that IBI352g4a, a PVRIG antibody with full Fc function, elicits strong antitumor efficacy via NK cell activation both in vitro and in vivo. By conducting mechanistic in vivo studies, we clearly demonstrated the primary role of NK cells in the antitumor effect of IBI352g4a and the great contribution of FcrR-binding properties to the functional effects exerted by this anti-PVRIG antibody. Our results support the further development of IBI352g4a and demonstrate its advantage over other Fc-silenced or attenuated monoclonal antibodies.

The nectin and nectin-like families are composed of important molecules that belong to the immunoglobulin superfamily and are involved in regulating cell‒cell adhesion and interactions [14, 15]. Along with other coinhibitory receptors, CD96 and TIGIT, and the costimulatory receptor CD226 constitute a critical regulatory network for lymphocyte activity and antitumor immunity, with the shared ligands CD155 and PVRIL2. Like TIGIT, PVRIG, the main inhibitory receptor, binds to PVRL2 with a greater affinity than CD226. Blockade of the PVRIG/PVRL2 interaction with an anti-PVRIG antibody may result in a weakened inhibitory effect of PVRIG and a strengthened activating effect of CD226 [5].

Previous studies have demonstrated that PVRIG is highly expressed on activated T cells, especially on terminally exhausted CD8^+^ T cells. However, blockade of PVRIG restores T-cell proliferation and cytokine production [7, 12]. This phenomenon has also been observed in PVRIG knockout mice, in which tumor-infiltrated T cells exhibit enhanced IFN-γ and TNF-α production [7]. Moreover, Li et al. [8] demonstrated that in addition to CD8^+^ T cells, NK cells also play essential roles in antitumor immunity induced by anti-PVRIG blockade.

Consistent with the findings of previous reports [12, 16], in our study, PVRIG was found to be highly expressed on both activated T cells and NK cells. However, in in vitro functional assays, we only observed NK cell activation when the cells were treated with PVRIG blockade, and no significant T-cell activation or proliferation was detected in several assays. These data suggest that NK cell-mediated effects of IBI352g4a are obviously more prominent than those of T cells. These findings have also been reported in vivo. In humanized mice, IBI352g4a was effective in a mice tumor model with both NK and T-enriched humanized model, but showed limited efficacy in a T-cell-only constitution model. In the TIL analysis, NK cell activation was first detected after the 1st dose of the anti-PVRIG antibody, while significant T-cell activation was not observed until the 2nd dose. This unusual phenomenon may be due to activated NK cell-derived cytokines or chemokines directly activating or promoting nearby T cells and further enhancing their cytotoxic effects [17]. However, the efficacy of anti-PVRIG was abolished in mice tumor models when either T or NK cells have been depleted. These data supported that both NK and T cells contributes to the antitumor effect, while NK is more predominant than T cells. Additional studies are needed to fully understand the mechanism by which IBI352g4a regulates NK and T cells to achieve its antitumor effect.

Taken together, these data suggested that NK cells are the primary target cells of PVRIG blockade, while T-cell activation may be a secondary effect of the cytokines released from NK cells. Furthermore, the antitumor effect of PVRIG blockade was observed only in the both NK- and T-enriched PBMC-reconstituted xenograft model. These data also confirmed the importance of NK cells in anti-PVRIG-induced antitumor efficacy and tumor immunity.

In addition to the activation effect on NK cells, we also observed that the antitumor effect of IBI352g4a was Fc dependent. Several studies have demonstrated that several immunomodulatory antibodies, such as GITR, OX40, CTLA-4 and TIGIT, depend on full Fc function for optimal antitumor activity [11, 18–20]. The mechanisms by which Fc-mediated effector functions enhance the efficacy of immunomodulatory antibodies have been previously described. However, unlike CTLA-4 or TIGIT, the depletion of intratumoral Tregs may not be the mechanism underlying the Fc-dependent antitumor effect of IBI352g4a. In our case, the optimal efficacy may be attributed to the stronger NK cell activation induced by FcγR coengagement. CD16a (FcγRIIIA), which has a higher affinity for IgG1 than for IgG4, is the dominant FcγR expressed on NK cells [21]. Coengagement of PVRIG and CD16a in NK cells leads to increased NK cell activation and improved efficacy. These data suggest that different NK activation pathways, such as the DNAM-1 and CD16a pathways, may amplify downstream pathways, resulting in stronger signals.

In addition to the coengagement of different pathways on NK cells, Fc-dependent activation of myeloid cells may be another mechanism underlying the optimized efficacy of IBI352g4a. As previously reported, PVRIG is more highly expressed in the tumor microenvironment than in the periphery, and abundant FcγR-expressing myeloid cells infiltrate tumors, creating a highly immunosuppressive environment [22, 23]. Thus, these findings create an ideal scenario for FcγR-mediated myeloid cell activation via the combination of anti-PVRIG antibody with an Fc-competent antibody, leading to optimal immune stimulation. Additionally, it has been well documented that IgG complex-induced ITAM-bearing type I FcγRs lead to substantial cellular activation and effector functions, such as phagocytosis, dendritic cell maturation, antigen presentation and macrophage polarization [24–26]. However, additional studies are needed to fully elaborate how IBI352g4a modulates the tumor microenvironment via FcγRs. To our knowledge, this is the first report showing that the antitumor efficacy of an anti-PVRIG antibody directly relies on Fc function and NK cell activation. We clearly showed that the anti-PVRIG treatment was ineffective if the xenografts lacked NK cells. However, the increase in NK cells after coengagement of IBI352g4a and FcγR led to improved efficacy.

In summary, our study demonstrated the antitumor effect and tumor immune activation of the anti-PVRIG mAb IBI352g4a in preclinical studies. Our data highlighted that both NK and T cells contribution to the antitumor efficacy induced by PVRIG antibody, but NK cells mediated effects are more prominent than those mediated by T cells. Moreover, wild-type IgG1 Fc is required for optimal antitumor efficacy. Further studies demonstrating how anti-PVRIG agents combined with wild-type IgG1 modulate the tumor microenvironment, leading to improved efficacy, are required to fully understand the underlying mechanism involved.

### Supplementary Information

Below is the link to the electronic supplementary material.

Supplementary file1 (DOCX 742 kb)

## Abbreviations

CMV: Cytomegalovirus
CTV: Cell Trace Violet
IBI352g4a: A fully human anti-PVRIG therapeutic antibody
NK: Natural killer
PVRIG: Poliovirus receptor-related immunoglobulin domain-containing protein
TIGIT: T cell immunoreceptor with Ig and ITIM domains
PVR: Poliovirus receptor
PVRL2: Poliovirus receptor-related 2
PBMCs: Peripheral blood mononuclear cells
TIL: Tumor-infiltrating lymphocyte
