# Supplementary material for: Characterization of a novel anti-PVRIG antibody with Fc-competent function that exerts strong antitumor effects via NK activation in preclinical models
Source: Cancer Immunol Immunother. 2024 Mar 30;73(5):81. doi: 10.1007/s00262-024-03671-z (PMC10981589; doi:10.1007/s00262-024-03671-z)
Supplement: Supplementary file 1 — Supplementary file1 (DOCX 742 kb) [file 262_2024_3671_MOESM1_ESM.docx]

**Supplemental Material**

**Supplemental Figure 1**


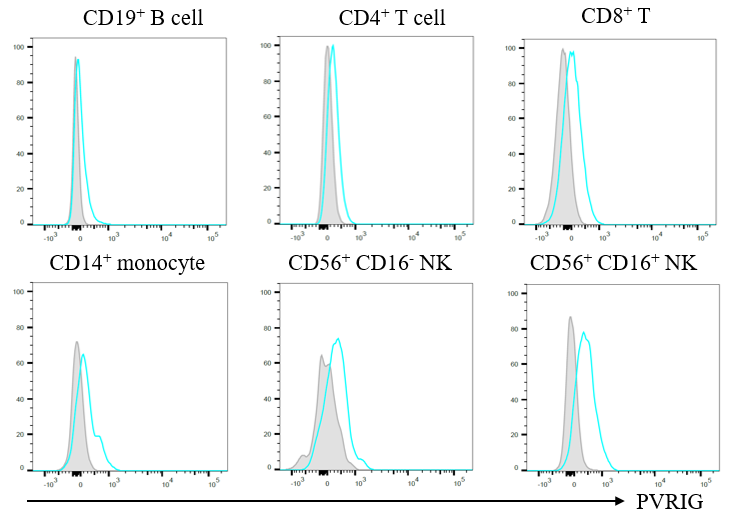


**Figure. S1** The expression of PVRIG on rested PBMC. Thaw PBMC, washed and resuspended with media (RPMI-1640 + 10% fetal calf serum). PBMC culture without stimulation for 20 hours and then detected the PVRIG expression on different cell subpopulations in PBMC by flow cytometry.

**Supplemental Figure 2**


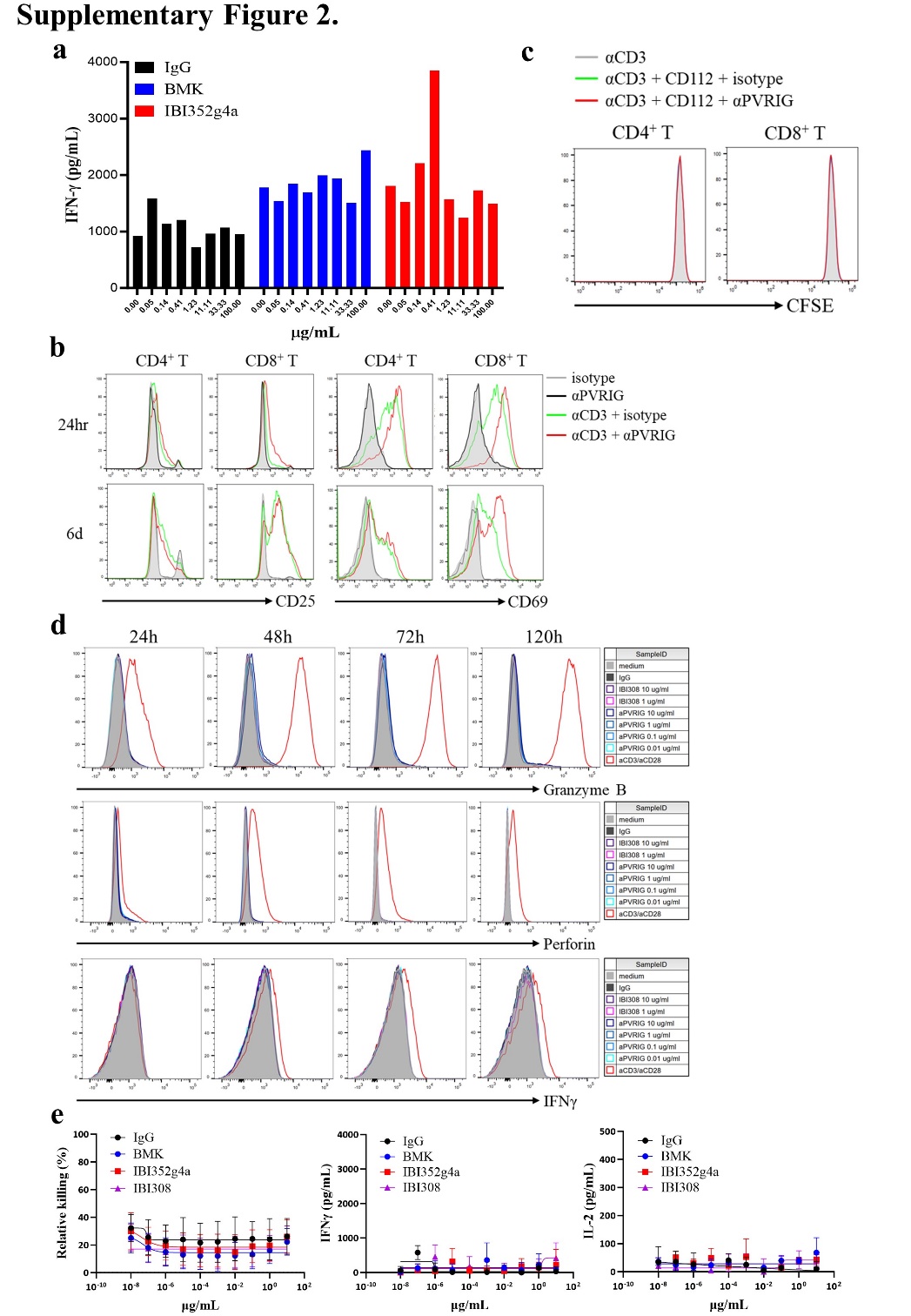


**Figure. S2 Anti-PVRIG antibody little induce T cells response.** a. PVRL2 precoated, and then PBMC stimulated with CMV in the increasing amounts of IBI352g4a or BMK. After 6 days of culture, secreted IFN-γ in the conditioned media by ELISA. b PBMC were stimulated with different relevant conditions. After 24 hour and 6 days of culture, CD25 and CD69 expression on CD4+T and CD8+T cell were analyzed by flow cytometry. c CFSE-labeled PBMC were stimulated with different relevant conditions. After 6 days of culture, cell division was analyzed based on the dilution of CFSE by flow cytometry. d T cells isolated from PBMCs by EasySepTM Human T cell Enrichment kit and were stimulated with different conditions. After 24 hours, 48 hours, 72 hours and 120 hours of culture, Granzyme B, Perforin and IFNγ on CD8+T cell were analyzed by flow cytometry. e Cytotoxicity and IFNγ and IL-2 release of T cells against K562 cells at 5:1 ration in the presence of different dose BMK, IBI352g4a or hIgG control antibody was analyzed.

**Supplemental Figure 3**


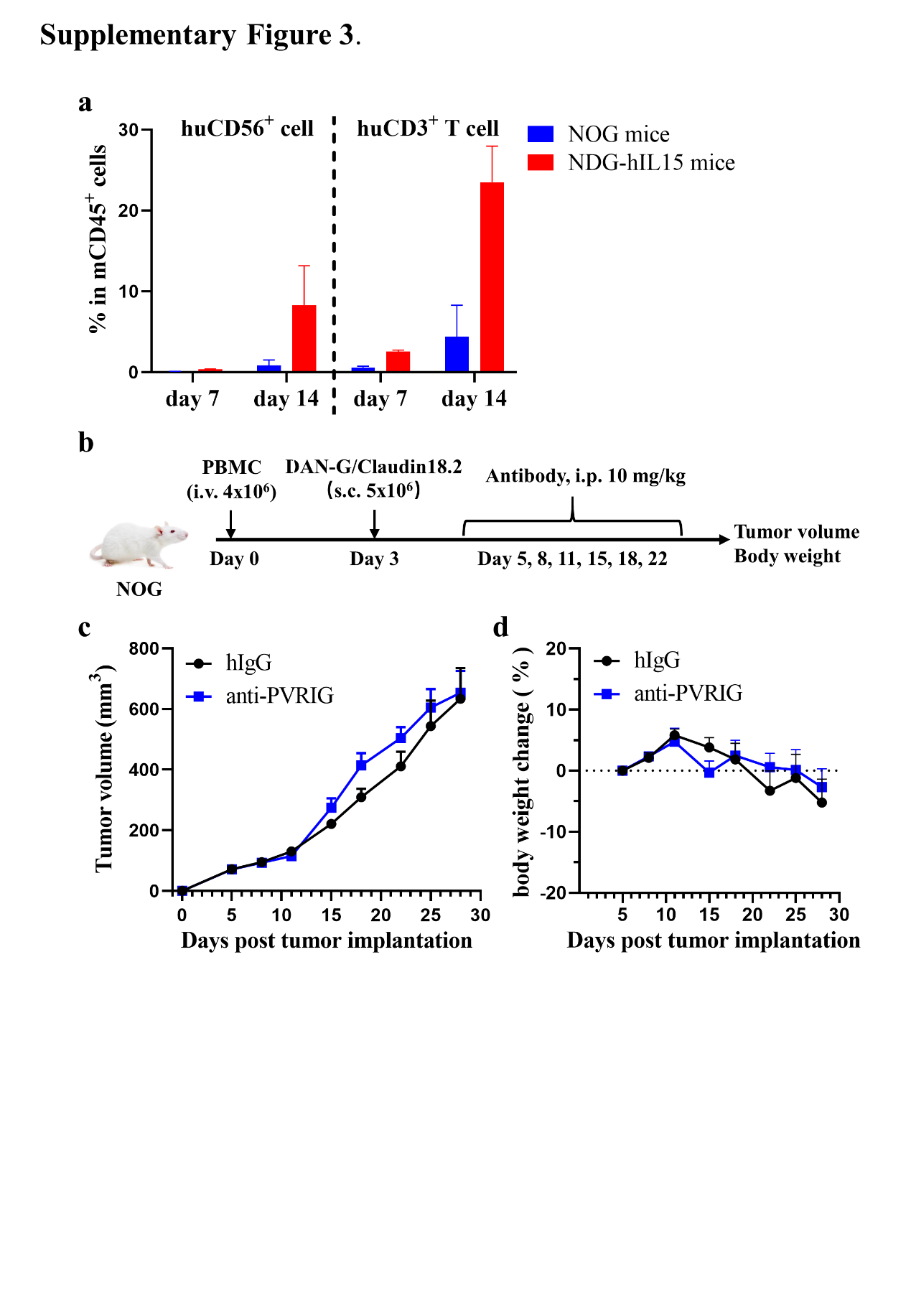


**Figure. S3** PVRIG blockade have no anti-tumor efficacy in humanized NOG mice bearing DAN-G/Claudin18.2. **a** NOG mice and NDG-hIL15 mice were injected intravenously (i.v.) PBMCs on day 0. Detected the human CD3^+^ T cells and human NK cells percentage in mouse CD45^+^ cells on day 7 and day 14 by flow cytometry. **b** NOG mice were injected intravenously (i.v.) PBMCs on day 0 and then inoculated subcutaneously with DAN-G/Claudin18.2 tumor cells on day 3. Mice were grouped randomly and then treated with hIgG (10 mg/kg) or anti-PVRIG (10 mg/kg) intraperitoneally (i.p.) twice one weekly starting on day 5 for six times. **c** Tumor size and **d** body weight change (%) of mice measured at various time points. Data show as mean + SEM.

**Supplemental Figure 4**


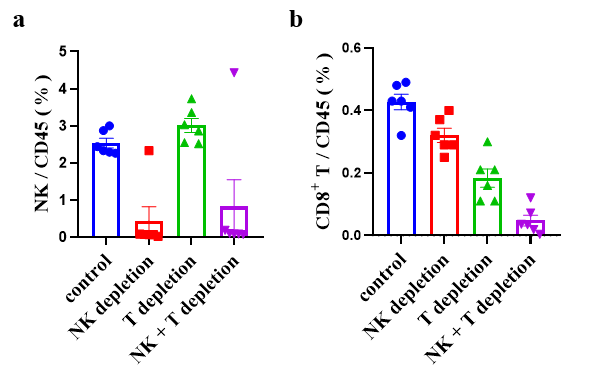


**Figure. S4** NK and T cell depletion efficiency. (**a**-**b**) Mice were intraperitoneally (i.p.) injected with PBS (control), 2.5mg/kg Asioa GM1 polyclonal antibody (NK depletion), InvivoMAb anti-mouse CD8a (Lyt3.2) (T depletion), 2.5mg/kg Asioa GM1 polyclonal antibody and 10mg/kg InvivoMAb anti-mouse CD8a (Lyt3.2) (NK + T depletion). Percentage of NK cells (**a**) and CD8^+^ T cells (**b**) in CD45^+^ cells in blood were analyzed by follow cytometry.
